# The Consequences of Chorioamnionitis: Preterm Birth and Effects on Development

**DOI:** 10.1155/2013/412831

**Published:** 2013-03-07

**Authors:** Robert Galinsky, Graeme R. Polglase, Stuart B. Hooper, M. Jane Black, Timothy J. M. Moss

**Affiliations:** ^1^The Ritchie Centre, Monash Institute of Medical Research, Monash University, P.O. Box 5418, Clayton, VIC 3168, Australia; ^2^Department of Obstetrics and Gynecology, Monash University, Clayton, VIC 3168, Australia; ^3^Department of Anatomy and Developmental Biology, Monash University, Clayton, VIC 3800, Australia

## Abstract

Preterm birth is a major cause of perinatal mortality and long-term morbidity. Chorioamnionitis is a common cause of preterm birth. Clinical chorioamnionitis, characterised by maternal fever, leukocytosis, tachycardia, uterine tenderness, and preterm rupture of membranes, is less common than subclinical/histologic chorioamnionitis, which is asymptomatic and defined by inflammation of the chorion, amnion, and placenta. Chorioamnionitis is often associated with a fetal inflammatory response. The fetal inflammatory response syndrome (FIRS) is defined by increased systemic inflammatory cytokine concentrations, funisitis, and fetal vasculitis. Clinical and epidemiological studies have demonstrated that FIRS leads to poor cardiorespiratory, neurological, and renal outcomes. These observations are further supported by experimental studies that have improved our understanding of the mechanisms responsible for these outcomes. This paper outlines clinical and experimental studies that have improved our current understanding of the mechanisms responsible for chorioamnionitis-induced preterm birth and explores the cellular and physiological mechanisms underlying poor cardiorespiratory, neural, retinal, and renal outcomes observed in preterm infants exposed to chorioamnionitis.

## 1. Preterm Birth

Preterm birth poses a major challenge for perinatal medicine, contributing to over 70% of perinatal mortality in developed countries (excluding deaths associated with congenital defects) [1–4]. Infants that survive preterm birth are more likely to suffer cardiorespiratory problems, mental retardation, cerebral palsy, and vision and hearing impairment, when compared to infants born at term [5]. 

Preterm birth is subcategorised according to gestational age at delivery: infants born preterm are delivered before 37 completed weeks of gestation. Late preterm births include infants delivered between 34 and 36 weeks and 6 days of gestation. Moderate preterm births are of infants delivered between 32 and 33 weeks and 6 days of gestation [4]. Infants born very preterm are delivered before 32 completed weeks; and infants born extremely preterm are delivered before 28 completed weeks of gestation [6].

Survival of extremely preterm infants has improved over the past decade, with the threshold of viability (defined as the gestational age at which 50% of infants survive) falling to less than 24 weeks [7]. Improved survival is a direct result of advances in perinatal care that include the use of antenatal glucocorticoids for precocious maturation of fetal organs, postnatal surfactant therapy for optimizing lung function, and the use of less injurious neonatal resuscitation strategies such as continuous positive airway pressure and noninjurious positive pressure ventilation [7–12].

The incidence of premature birth in developed countries varies from 7.6 to 12% of all births [13–15], whilst in many low-to-middle-income countries the incidence of preterm births is ≥15% of all births [14]: alarmingly, the incidence continues to rise [14, 15]. The World Health Organisation estimates there are 15 million preterm births globally and 1 million direct fatalities annually [4].

Data from the United States indicate that the annual cost of neonatal care for preterm babies is ~US$6 billion annually [10, 16], whilst the estimated societal economic impact is ~US$26.6 billion [17]. The cost of caring for individual infants varies according to their gestational age [18], with care for an extremely preterm infant reaching ~US$250,000 [10]. 

### 1.1. Etiology of Preterm Birth

There are a number of known contributors to premature birth [19], including the following:

- spontaneous preterm labour,
- multiple pregnancy,
- assisted reproduction,
- preterm prelabour rupture of membranes,
- hypertensive disorders of pregnancy (e.g., preeclampsia),
- intrauterine growth restriction,
- antepartum haemorrhage,
- miscellaneous (e.g., cervical incompetence, uterine malformations),
- *intrauterine inflammation/Chorioamnionitis.*

Intrauterine inflammation most commonly presents as chorioamnionitis, which is defined as inflammation (caused usually by bacterial infection) of the chorion, amnion, and placenta. Intrauterine inflammation is one of the most common antecedents of premature birth [20].

The incidence of intrauterine inflammation is inversely related to gestational age, such that it is implicated in the majority of extremely preterm births and 16% of preterm births at 34 weeks [21, 22]. Microbiological studies indicate that intrauterine inflammation is associated with approximately 25–40% of all preterm births [20, 23, 24]. This is likely to be a conservative estimate due to the difficulty associated with detecting chorioamnionitis using conventional culture techniques [20]. 

### 1.2. Chorioamnionitis

Chorioamnionitis may manifest as a clinical condition defined by maternal fever, leukocytosis, tachycardia, uterine tenderness, and preterm rupture of membranes [25, 26]. The diagnosis of clinical chorioamnionitis is most commonly made during labour near or at term. Highly virulent organisms likely cause clinical chorioamnionitis [27]. Before 30 weeks of gestation, clinical chorioamnionitis is usually diagnosed after attempting to delay preterm delivery or with preterm prolonged rupture of the fetal membranes [27].

Alternatively, chorioamnionitis can be subclinical, which is considered the most common manifestation and is defined histologically by inflammation of the chorion, amnion, and placenta [26, 28]. Histological chorioamnionitis is associated with organisms considered to be of low virulence. Deliveries prior to 30 weeks of gestation are typically associated with histological chorioamnionitis [22]. Histological diagnosis occurs after delivery and is based on a semiquantitative assessment of inflammatory cells in the chorioamniotic membranes, umbilical cord (in cross-section), and placental disc. However, variability in the assessment criteria for the diagnosis of histological chorioamnionitis exists within the literature [29]. This may influence the results of studies of histological chorioamnionitis, preterm delivery, and outcomes.

The majority of fetuses exposed to chorioamnionitis develop a systemic inflammatory response known as the fetal inflammatory response syndrome (FIRS) [30, 31]. This is due to the fetus being in direct contact with infected amniotic fluid and/or inflammatory cell transfer from the uteroplacental circulation. FIRS can itself be categorised as clinical or subclinical. Clinical FIRS is defined by a fetal plasma [interleukin-6] >11 pg/mL [32], whilst subclinical FIRS is defined histologically by funisitis and fetal vasculitis [31].

### 1.3. Animal Models of Chorioamnionitis/Intrauterine Inflammation

Intrauterine inflammation can be produced in experimental animals by exposing the fetus to lipopolysaccharide (LPS), derived from the outer cell wall of gram-negative bacteria. LPS is capable of inducing an inflammatory cascade (which is the dominant feature of clinical and subclinical chorioamnionitis), in the absence of bacterial infection. 

Intracervical LPS administration to pregnant rats and rabbits has been used to model clinical chorioamnionitis [33, 34]. Injection of LPS into the cervix causes high-grade placental inflammation [33, 34] associated with a maternal systemic inflammatory response that extends to the fetus and causes moderate to high rates of fetal loss [34–37]. The consequences of this experimental intervention mimic the most severe forms of clinical chorioamnionitis.

Other models of clinical chorioamnionitis include intravenous and intraperitoneal administration of LPS to pregnant animals. Intra-venous administration of LPS to pregnant sheep causes maternal pyrexia, septicaemia, and increased uterine contractility [38]. The effects on the fetus include systemic inflammation, increased serum cortisol levels, preterm delivery, and death [38, 39]. Direct intravenous administration of LPS to fetal sheep causes a high-grade FIRS that results in almost a 50% premature death rate [40]. In mice, maternal intravenous LPS exposure causes systemic and placental inflammation and altered placental vascular function [41, 42]. Fetal demise due to administration of LPS to pregnant mice is dose dependant [42]. 

Intraperitoneal injection of LPS to pregnant rodents elicits a maternal systemic inflammatory response that causes placental inflammation, FIRS [43–45], and, in some cases, fetal death [46]. Interestingly, rats seem to be more resistant to inflammation-induced preterm birth than mice [46].

In sheep, rabbits and rodents, subclinical chorioamnionitis/intrauterine inflammation can been induced by injecting LPS into the amniotic cavity [47–49]. This does not present with clinical symptoms in the pregnant animal and causes a low-grade FIRS that is usually tolerated without fetal demise [47, 50, 51]. 

### 1.4. Microbial Invasion of the Amniotic Cavity

Microbiota may invade the amniotic cavity via several pathways, outlined previously [20, 23, 52]. The most common mode of invasion involves microbes ascending from the vagina and/or cervix, resulting in an initial restricted invasion of the amniotic cavity. Microbes proliferate in the amniotic fluid and subsequently invade the amnion. In severe cases choriodecidual invasion may occur [53]. Thus, microbial invasion of the amniotic cavity precedes widespread infection of the chorioamniotic membranes. Other modes of microbial invasion of the amniotic cavity include contamination during invasive obstetric procedures such as amniocentesis or chorionic villous sampling; haematogenous dissemination through the placenta; and retrograde invasion from the peritoneal cavity through the fallopian tubes. 

Traditionally, the microorganisms most commonly associated with infection of the amniotic cavity were species of *Ureaplasma* and *Mycoplasma* such as *Ureaplasma urealyticum*, *Ureaplasma parvum, *and* Mycoplasma hominis* [54, 55]. Due to recent advances in microbial detection, the range of microbial colonies in the amniotic cavity is now regarded as more diverse. *Fusobacterium, Sneathia,* and *Leptotrichia *have each been identified as novel and highly prevalent bacterial antecedents of chorioamnionitis [56, 57]. Furthermore a distinct difference in the prevalence/distribution of bacterial taxa exists between women that deliver preterm with intact membranes versus women that deliver preterm with premature rupture of membranes [56]. 

Whilst there is a wide range of data describing the role of bacterial invasion of the intrauterine space in premature labour, data describing the role of viruses and fungi in premature labour are limited. There is some evidence demonstrating viral and fungal invasion of the amniotic cavity in the pathogenesis of intrauterine inflammation. Specifically, cytomegalovirus, parvovirus, adenovirus, and the fungal phenotype *Candida albicans* have been detected in amniotic fluid samples [58–60]. There are data to suggest pregnant women with hepatitis B virus are at increased risk of premature labour [61, 62]. Furthermore, intrauterine injection of polyinosinic-cytidylic acid (Poly I:C), a viral mimetic, causes preterm birth in rodents [63], whereas no effects of intra-amniotic Poly I:C were observed in sheep [64].

### 1.5. Chorioamnionitis Causes an Inflammatory Cascade That Leads to Preterm Birth

Preterm labour may result from a fetal and/or maternal response to chorioamnionitis. Bacteria that invade the choriodecidual space release endotoxins and exotoxins, which are recognised by Toll-like receptors (TLRs) on the surface of leukocytes, and dendritic, epithelial, and trophoblast cells [65, 66]. This activates transcription factors NF-*κ*B, AP-1, and STAT which produce cytokines and chemokines such as interleukin (IL)-6, IL-1*α*, IL-1*β*, IL-8, and tumour necrosis factor-*α* (TNF *α*) within the decidua and the fetal membranes [23]. Inflammatory cytokines stimulate the production of prostaglandins and initiate neutrophil chemotaxis, infiltration, and activation, resulting in the synthesis and release of metalloproteases [54]. Prostaglandins stimulate uterine contractions while metalloproteases cause cervical ripening and degrade the chorioamniotic membranes causing them to rupture [67]. 

Prostaglandins produced in the amnion are normally inactivated by prostaglandin dehydrogenase released by the chorionic tissue, thus preventing prostaglandins reaching the myometrium and causing uterine contractions [23]. Infection of the chorion inhibits the activity of prostaglandin dehydrogenase, thereby allowing prostaglandins to reach the myometrium and cause premature contractions [68]. 

In human pregnancies affected by chorioamnionitis, FIRS increases the production of corticotrophin releasing hormone (CRH) from both the fetal hypothalamus and the placenta [23]. Increased CRH causes the fetal adrenal glands to increase cortisol production, which stimulates placental prostaglandin synthesis and myometrial contractility [69]. However, in studies using a sheep model of histological chorioamnionitis that displays many characteristics of the human condition, Nitsos et al. [51] showed that increases in fetal cortisol levels are minimal and unlikely to impact on fetal development.

### 1.6. Chorioamnionitis Affects Multiple Organ Systems

Chorioamnionitis, together with the associated FIRS, is an antecedent of preterm labour and a major contributor to neonatal morbidity [23, 54, 55, 70, 71]. The consequences of intrauterine inflammation on fetal and neonatal cardiopulmonary, cerebral, and renal systems are described in the following sections.

#### 1.6.1. Heart

Evidence from humans and experimental models of chorioamnionitis suggest intrauterine inflammation results in abnormal fetal cardiac function. In humans, intrauterine inflammation has been associated with increased left ventricular compliance/dilatation [72]; this is thought to be a compensatory mechanism for maintaining left ventricular output and is commonly observed in adult patients experiencing sepsis [73]. Human neonates born after exposure to inflammation/infection *in utero* exhibit reduced mean and diastolic blood pressure, effects which may contribute to the increased incidence of periventricular leukomalacia and cerebral palsy in neonates exposed to chorioamnionitis *in utero* [74]. 

These clinical observations are supported by animal studies that have demonstrated reduced descending aorta blood flow velocities in fetal rats following intracervical administration of LPS to pregnant dams [33]. In fetal mice intra-amniotic LPS exposure caused inflammation and impaired contractility and relaxation of the myocardial tissue [75]. Increased cardiac afterload and reduced cardiac output have also been observed in fetal mice after maternal LPS administration [41]. Abdulkadir et al. [33] demonstrated that chorioamnionitis induced by intracervical LPS exposure caused a reduction in heart rate in newborn rat pups. *In vitro* studies have demonstrated that treatment of immature cardiomyocytes with LPS for 72 hours stimulated the production of inflammatory molecules within the cardiomyocytes and caused cardiomyocyte loss that was reversible with NF-*κ*B inhibition [76]. Collectively these studies indicate that exposure to inflammation *in utero* not only impairs cardiac function but may also impair development of the myocardium, with likely long-term deleterious consequences. Given the evidence from human and animal studies, investigation of the long-term cardiovascular consequences of exposure to intrauterine inflammation is warranted.

#### 1.6.2. Lungs

In 1996, Watterberg et al. [77] demonstrated that infants exposed to chorioamnionitis had a reduced risk of developing respiratory distress syndrome (RDS) and an increased risk of bronchopulmonary dysplasia (BPD). RDS is caused primarily by a lack of pulmonary surfactant and its incidence is inversely associated with gestational age at delivery [10]. It is characterised by tachypnoea, chest wall retraction, cyanosis, and a ground glass appearance of the chest on X-ray [78].

BPD is defined as the need for supplemental oxygen beyond one month of postnatal age [79, 80] and is most commonly observed in extremely preterm infants [79]. BPD is characterised by impaired vascularisation and alveolarization of the developing lung, whereby pulmonary microvascular angiogenesis is disrupted and alveoli are fewer in number and larger in size [27, 80]. An increased risk of BPD in preterm neonates exposed to chorioamnionitis may be mediated by other postnatal events such as mechanical ventilation and oxygen exposure [80]. 

Since the initial description by Watterberg et al. [77], clinical data have accumulated to demonstrate that the relationship between chorioamnionitis, RDS, and BPD is more complex than initially described. Been et al. [81] demonstrated that infants diagnosed with chorioamnionitis and FIRS had an increased risk of developing RDS and were less responsive to surfactant treatment relative to infants diagnosed with chorioamnionitis but without FIRS. In contrast, infants diagnosed with chorioamnionitis but without accompanying FIRS had less severe RDS than infants who were not exposed to chorioamnionitis. Recent studies have been unable to demonstrate an independent association between chorioamnionitis and the development of BPD in preterm infants [82, 83], likely because of the complex variety of prenatal inflammatory stimuli and their interaction with ventilatory management of neonates [84].

Animal studies clearly indicate that fetal lung development is altered by intrauterine inflammation. In rats, intra-amniotic administration of inflammatory cytokines such as IL-6 and IL-8 increases the expression of messenger RNA for surfactant proteins (SP) A, B, and C in the fetal lung [85]. The increase in surfactant protein production is thought to be associated with an increase in type 2 alveolar epithelial cells because *in vivo* and *in vitro* studies of fetal mice show that intra-amniotic LPS exposure increases type 2 alveolar epithelial cell numbers [86]. Consistent observations have been made in rabbits and sheep whereby intra-amniotic or intratracheal injection of IL-1*α* or LPS resulted in increased mRNA expression of SP-A and -B in the fetal lungs [87–90]. We have recently demonstrated that the fetal lung response to intrauterine inflammation is mediated, at least in part, by prostaglandins [90].

In sheep, intrauterine inflammation causes pulmonary inflammation with increased mRNA levels of inflammatory cytokines IL-1*β*, IL-6, and IL-8 and chemokines IP-10 and MIG within 24 hours [50, 91]. Reduced expression of microvascular markers (vascular endothelial growth factor (VEGF), VEGF receptor 2, endothelial nitric oxide synthase (eNOS), tyrosine protein kinase receptor (Tie-2), and platelet endothelial cell adhesion molecule (PECAM)) occurs between 1 and 4 days after LPS exposure [92]. 

Inflammation-induced alterations to fetal pulmonary vascular development include smooth muscle hypertrophy and deposition of collagen in the adventitial layer of pulmonary resistance arterioles [92]. We have recently observed that these alterations to the pulmonary vasculature are associated with an increase in pulmonary vascular resistance and subsequent reduction in pulmonary blood flow in the fetus at 2 and 4 days, respectively, after intra-amniotic LPS exposure [93].

Structural remodeling of the airspace occurs 7 days after intra-amniotic injection of LPS in sheep, resulting in the presence of fewer and larger alveoli (20% decrease in alveolar number and 30% increase in alveolar volume), and thinning of the alveolar epithelial layer [88].

One of the postnatal consequences of inflammation-induced vascular remodelling of the fetal lungs is persistent pulmonary hypertension of the newborn (PPHN). This condition is characterised by increased resistance to pulmonary blood flow and right-to-left shunting across the foramen ovale (FO) and ductus arteriosus (DA), resulting in decreased left ventricular output [94, 95]. PPHN increases the risk of BPD and hypoxemia, but is also increased by BPD [96]. Preterm lambs exposed to a single injection of intra-amniotic LPS 7 days prior to delivery showed increased pulmonary vascular resistance and right-to-left shunting of blood through the DA within 30 minutes after delivery [97]. LPS exposure 2 or 4 days prior to delivery does not have such profound effects on pulmonary haemodynamics of preterm lambs, suggesting that the full extent of vascular remodelling had not occurred by that time [98]. Considering the pulmonary vascular and alveolar remodelling demonstrated in fetal lambs exposed to intra-amniotic LPS [88, 92], a causative link between chorioamnionitis, BPD, and PPHN becomes increasingly apparent.

#### 1.6.3. Brain

In preterm infants, perinatal brain damage is a major cause of developmental delay and lifelong neurological impairments such as mental retardation, cerebral palsy, and learning, and behavioural deficits [99–101]. In the US, the estimated lifetime costs for persons born with mental retardation is $51.2 billion and $11.5 billion for persons born with cerebral palsy [102]. There is robust epidemiological evidence linking perinatal brain injury, in particular cerebral palsy, periventricular leukomalacia, and intraventricular haemorrhage, with intrauterine inflammation [25, 103–108]. Exposure to histological chorioamnionitis combined with impaired placental perfusion has been demonstrated to increase the risk of poor neurological and neurocognitive outcomes at 2 years of corrected age in children born very preterm [99]. Similar observations were made at 8 years of age in children exposed to severe histological chorioamnionitis [100]. Histological chorioamnionitis is also associated with an increased incidence of speech delay and hearing loss at 18 months of corrected age in infants born very preterm [101]. Furthermore, histological chorioamnionitis caused by bacterial and viral infection has been associated with an increased risk of autism spectrum disorders [109] and schizophrenia [110, 111]. Recent work suggests that persistent inflammation is responsible for phenotypic abnormalities observed in autism, whereas a latent inflammatory process *in utero* appears responsible for schizophrenia-specific brain and behavioural abnormalities [112]. 

A number of clinical studies have identified potential mechanisms for associations between chorioamnionitis and adverse neurological outcomes. A direct effect of immune activation is demonstrated by studies showing that intrauterine inflammation is linked with diffuse white matter injury in the brain of preterm neonates, due to activation of a systemic inflammatory cascade [99, 105–107, 113]. 

Chorioamnionitis has been associated with impaired fetal and newborn cardiac function [74, 114], which may compromise brain blood flow. Lower blood pressures and higher concentrations of inflammatory mediators have been demonstrated in the systemic circulation in very low birth weight infants exposed to chorioamnionitis [107, 115, 116]. Yanowitz et al. [117] demonstrated that cerebral oxygen delivery is altered in prematurely born infants exposed to chorioamnionitis. These observations suggest that cerebral blood flow and/or autoregulation of cerebral blood flow are impaired in infants born following exposure to chorioamnionitis. 

Impaired cerebral autoregulation is considered one of the main contributors to brain injury in the preterm neonate [118, 119] and has previously been demonstrated in preterm infants during the first 120 hours after birth [120, 121]. Impaired cerebral autoregulation may be more prevalent in neonates born after exposure to intrauterine inflammation [115, 119, 122, 123]; however there are limited data that directly support this contention. 

Data from animal experiments are consistent with human studies in showing effects of intrauterine inflammation on the developing brain. Rabbit pups exposed to a single intra-amniotic injection of *E. coli* showed periventricular lesions documented in the form of karyorrhexis (nuclear fragmentation) of glial cells and reduced density and disorganization of white matter [124]. In fetal sheep, chronic administration of intra-amniotic LPS derived from *E. coli* resulted in damage to subcortical white matter in the form of astrocytosis and a reduction in oligodendrocyte number [125]. Intravenous administration of LPS to fetal lambs caused diffuse damage and focal PVL in white matter [126]. In the offspring of pregnant rats exposed to a single intraperitoneal injection of LPS, a decrease in myelination was observed, potentially due to reduced numbers and/or function of oligodendrocytes [127]. 

Recent experiments examining the effect of intrauterine inflammation on cerebral haemodynamics have shown that within 15 minutes after delivery carotid arterial blood flow and pressure are increased in preterm lambs 2 days after LPS exposure [98]. Additionally, carotid arterial pressure was shown to be increased 1 hour after preterm delivery of lambs 7 days after LPS exposure [97]. Such disturbances in cerebral haemodynamics may increase susceptibility to brain injury in the preterm neonate.

We have demonstrated an increase in inflammatory cytokine mRNA expression in the periventricular and subcortical white matter and periventricular vascular damage and haemorrhage, 48 to 96 hours after exposure to intra-amniotic LPS [98], and increased cerebral perfusion after 4 and 5 days in preterm fetal sheep [93]. This finding is consistent with the observation that cerebral DO_2_ is increased in preterm fetal sheep exposed to intra-amniotic LPS, indicating an increased cerebral metabolic demand before birth [128].

Inflammatory cytokines released during the course of intrauterine inflammation have been suggested as a possible cause of cerebral injury observed in animal studies [129, 130]. The potential mechanisms of inflammatory cytokine induced brain injury include the following:

1. a direct effect on the cerebral vasculature causing cerebral hypoperfusion and ischemia [74, 131],
2. activation of blood coagulators resulting in capillary thrombosis and necrosis of white matter [132],
3. activation of microglia, causing a direct toxic effect on oligodendrocytes and myelin via microglial production of proinfammatory cytokines, neuronal loss and impaired neuronal guidance [133–135]. Microglial activation also generates free radicals that cause death of immature oligodendrocytes [108, 136],
4. increased permeability of the blood brain barrier, allowing direct passage of microbial products and cytokines into the cerebral tissue [137–139].

#### 1.6.4. Retina

Recent evidence suggests an association between chorioamnionitis and retinopathy of prematurity (ROP). Higher rates of ROP have been demonstrated in infants born to mothers with histological and clinical chorioamnionitis relative to mothers without chorioamnionitis [140–142]. Chorioamnionitis and the accompanying fetal inflammatory response syndrome may increase the risk of ROP by directly sensitising the developing retina to oxygen-induced changes in VEGF availability and subsequent vascular development and/or by causing systemic hypotension resulting in retinal hypoperfusion/ischemia [143, 144]. Frequent, intermittent hypoxic events are associated with severe ROP [145]. These clinical data indicate a need for experimental studies to elucidate the pathophysiological mechanisms underlying the increased risk of ROP in infants exposed to chorioamnionitis.

#### 1.6.5. Kidneys

Clinical data demonstrating an effect of chorioamnionitis on the developing kidney are limited. In a study of women with preterm premature rupture of membranes, FIRS was associated with oligohydramnios [146]. Since fetal urine production is a major component of amniotic fluid volume, this suggests a reduction in fetal renal function, perhaps as a consequence of redistributed blood flow away from the fetal kidneys [146]. Similar observations have been made in adult patients suffering from septicaemia, where oliguria is thought to be a manifestation of renal inflammation [147, 148]. Chorioamnionitis has been associated with renal and electrolyte abnormalities in preterm neonates treated with indomethacin, suggesting chorioamnionitis may adversely affect renal development [149]. 

Experimental evidence from our laboratory has demonstrated that preterm fetal sheep exposed to intrauterine inflammation have a reduction in nephron number of ~20% [150]. This may predispose the preterm infant exposed to chorioamnionitis to impaired renal function during the neonatal period and an increased risk of hypertension and renal dysfunction later in life, in accordance with the Brenner hypothesis [151]. At this point in time the effects of intrauterine inflammation on renal development and function and how it leads to a reduction in the number of nephrons formed in the kidney are largely unknown. Further studies are required to determine the mechanisms underlying the inflammation-induced reduction in nephron number and the extent of the renal and cardiovascular consequences in the neonate and adult. 

## 2. Summary

Available clinical, epidemiological, and experimental data indicate that chorioamnionitis plays a significant role in predisposing the preterm infant to multiple organ disease. Further investigation is required to improve our understanding of the mechanism(s) underlying the changes in development and function of the preterm cardiorespiratory, central nervous, visual, and renal systems. Improved antenatal screening for chorioamnionitis and identification of effective treatment strategies for preterm infants exposed to intrauterine inflammation will likely provide a better prognosis for infants at risk of multiple organ disease as a result of exposure to inflammation before birth.
